# Evaluating the Validity of Model Organisms: A Review and a New Framework for Biologists

**DOI:** 10.1096/fj.202600104R

**Published:** 2026-08-29

**Authors:** Héloïse Athéa, Nicolas Heck

**Affiliations:** ^1^ Inserm, Gustave Roussy Institute, Université Paris‐Saclay Villejuif France; ^2^ Institut d'Histoire et de Philosophie des Sciences et des Techniques Paris France; ^3^ Sorbonne Université, CNRS, INSERM, Center for Neuroscience Sorbonne University Paris France

## Abstract

Model organisms are central to biological research. Living organisms are models in the sense that the knowledge gained from their study is meant to be generalized. For example, experiments conducted on *Drosophila* or nematodes bring general knowledge on phenomena such as cell migration, studies in zebrafish or mice allow researchers to find therapeutical approaches applicable to human diseases, and results obtained from the study of Arabidopsis are generalized to the plant kingdom. Furthermore, new organisms gain the status of models, and non‐traditional model organisms provide new perspectives on biological processes. However, the extent to which results can be generalized, or external validity, mostly relies on assumptions. Herein, a conceptual analysis is developed to justify the generalization of knowledge from one model organism to other species or human clinical contexts. After summarizing the concept of model as stated in philosophy of science, the specific features of model organisms are presented. The existing arguments put forward to justify the external validity of model organisms are then reviewed. In the second part of this article, a new framework is proposed that sets a system of criteria to assess model organisms' external validity. While grounded in concepts of philosophy of science that define the conditions under which empirical knowledge is valid, this framework is designed for use by biologists.

## Graphical abstract

This article provides an overview on model organisms and proposes a new framework for assessing the extent to which it is justified to generalize the results obtained from a given organism. It covers both studies of human diseases and those focused on general biological processes. The first part of the article is a review on model organisms. It is followed by a review of existing proposals that have been put forward to assess the validity of living organisms as models. The second part presents a new framework that allows biologists to assess their model organism's validity. It is developed on a comprehensive approach that combines rigorous philosophical criteria with practical guidelines applicable across diverse biological disciplines. The proposed evaluative framework illustrates how philosophy of science can clarify concepts and structure critical thinking on experimental methods and scientific assumptions.

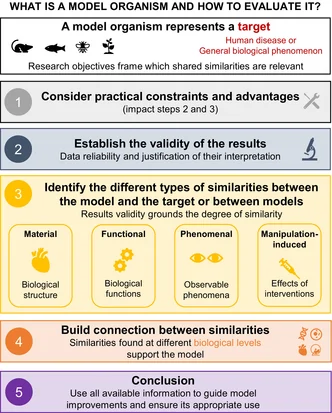

## 1 Introduction

From basic questions about biological processes to the testing of therapeutic hypotheses in biomedicine, model organisms (MOs) are fundamental to biological research. Living organisms are models in the sense that knowledge gained from studies conducted on them is assumed to generalize to other living organisms. Whether this assumption is justified is thus a central question. Herein, we review existing solutions and propose a new framework of criteria for assessing the validity of MOs. First, we offer a concise review of the concept of “model” as defined in the philosophy of science. In turn, we present the distinctive features that set living organisms apart from other forms of scientific modeling. Importantly, this review of general concepts and specific features guides the further discussion on the epistemic validity of MOs. We then examine proposals and arguments that have been put forward to justify that knowledge gained from MOs can be generalized to other species or transferred to humans. Finally, we present a new system that allows us to evaluate the epistemic validity of MOs. Whilst based on concepts of philosophy of science, this framework offers clear guidelines for biologists. Throughout this review and in developing the proposed framework, examples from biology are given, not only to make philosophical concepts accessible, but also to illustrate how the validity of the knowledge gained through biological research conducted with MOs stems from these concepts.

## 2 What Is a Model Organism?

### 2.1 What Is a Scientific Model?

Developing a framework to evaluate the validity of a MO first requires defining what a model is. While a thorough philosophical inquiry into the nature of models is beyond the scope of this paper, we will summarize key concepts in order to identify relevant criteria to assess the validity of MOs. At its core, a model can be defined as a representation [1]. This definition applies broadly across different types of models, including those representing theories and those depicting aspects of the world. Although seemingly simple, this definition and the notion of representation raise complex philosophical issues [2, 3]. Nevertheless, for our purposes, we adopt the widely accepted view that a model is a construction designed to represent a target—the target being the object or phenomenon under study. A classical account holds that models represent their targets through analogy. Accordingly, for a model to be justified, it must share similarities with its target. Many philosophers have attempted to provide a general account of similarity, but any two objects share countless similarities and differences. As a result, formulating a universal definition of models based on similarity may be hopeless [4]. Instead, what determines the validity of a model is its relevant similarity to its target [1]. A scientist, or a scientific community, uses a model to represent some aspects of the world for specific purposes [3]. Thus, the validity of a model must be assessed in relation to its adequacy for a given scientific purpose [5]. This principle is crucial for MOs: there is no such thing as a universally valid model, but rather models that are valid for particular research questions or hypotheses. Considering the purpose of a model is particularly important because models do not provide exact replications of reality: they are idealized representations. Idealization is an activity by which the modeler introduces simplifications, ignoring some variables and interactions between variables. The purpose of idealization may be to make a complex system more understandable, or to simplify the reality by retaining only the variables involved in the mechanism causing a particular phenomenon. Another form of idealization involves constructing multiple simplified models based on different assumptions, with each model capturing distinct aspects of the target [6]. It is noteworthy that idealization is an activity of the modeler; it does not include ignorance of variables due to limited knowledge of the target.

However, models do not merely represent: they are also investigative tools. Their design allows for manipulation or intervention, enabling scientists to learn about the target system [7]. In other words, a model is constructed to be both a representation of the target and an experimental setup that facilitates manipulations, measurements and observations. Representation and intervention are therefore intertwined, such that models are both “models of” and “models for” [8, 9]. Indeed, the model sets the conditions under which the phenomenon is realized, as well as the variables that can be manipulated and measured [10]. On one hand, the model shapes the kinds and extent of interventions and measurements that can be done; on the other hand, possible observations and interventions frame the representation. Because the construction or, in the case of MOs, the choice of the model, is guided by the study's objective, decisions about which parameters and properties of the model are relevant for the study shape both the conditions under which the model represents the phenomenon and how experiments are designed to produce meaningful data to study the phenomenon [11]. Consequently, a MO represents a target through shared similarities that are relevant for the specific scientific objectives. Evaluating its validity requires assessing whether idealization affects the trustworthiness of the results, and the extent to which it enables meaningful experimental manipulations and reliable measurements.

### 2.2 Representational Target and Scope of Model Organisms

**FIGURE 1:**
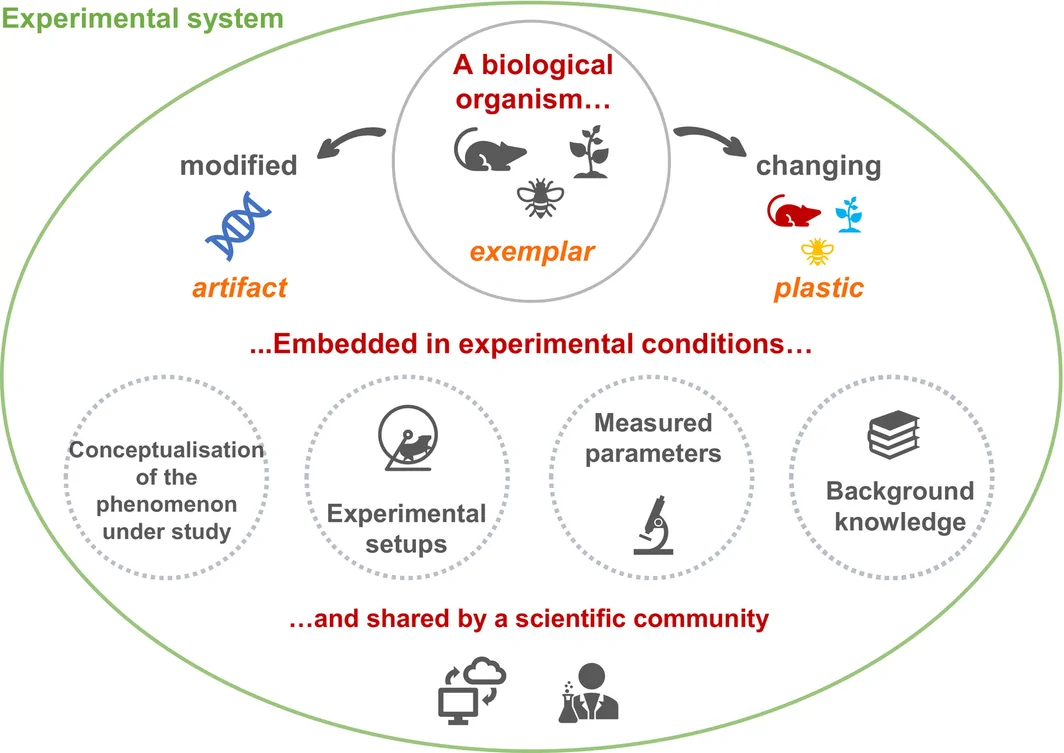
Model organisms are scientific models that have specific features. Living organisms can be used as models to represent targets. Model organisms are both exemplars and artifacts, and they possess plastic properties. The choice of controlled variables and measured parameters implies that model organisms are idealized experimental systems. The research objectives and background knowledge contribute as well to frame model organisms as experimental systems that are used by a scientific community. The knowledge and tools shared by the scientific community enable the establishment of the model and shape its use.

Beyond the general idea that a model represents a target, philosophers of science have argued that MOs exhibit distinctive features compared to other types of models (Figure 1). We identify six features that should be considered, as they either contribute to or constrain the validity of MOs (Box 1).

BOX 1 **The six features of model organisms.**

1. A wide variety of representational targets, ranging from simple biological processes to human patients. A given target is typically explored through multiple models, each based on analogical reasoning.
2. A representational scope based on evolutionary justification. The use of MOs is justified by evolutionary considerations that make the generalization of results a priori plausible.
3. A particular mode of representation. Compared to other scientific models, the way of representing the world associated with these models is unique: the living organisms used are both exemplars of the biological world and artifacts, idealized models.
4. A self‐modifying model. Organisms used as models are not static exemplars, but they interact with and modify themselves when manipulated or placed under experimental conditions.
5. A dynamic experimental system. MOs are living organisms embedded in experimental and theoretical frameworks, and this experimental system evolves as research progresses.
6. Original practical aspects. Their use involves practices and a way of doing science that are unique to them.

#### 2.2.1 The Representational Targets of MOs

First, MOs present a wide variety of “representational targets.” For MOs, the target is what can be studied through the use of an organism [12, 13]. In preclinical studies, the target is either a disease or a human patient (Box 2). Another type of target is a phenomenon, or biological process. For example, zebrafish or *Drosophila* are models that represent cell migration. In this context, a MO can represent multiple phenomena across different projects, such as cell proliferation, gene regulation, or heart development. In physiology, a MO's target may be a specific function: lampreys are studied to understand locomotion, and cats for vision. The phenomenon instantiated in a MO can be considered a representation, in that it mediates a particular phenomenon towards general knowledge. This representation here relies on analogical reasoning: although the MO and its target are never fully identical, they share relevant similarities and properties. Notably, some authors distinguish between models of human disease, called “surrogates,” and models of general phenomena, called “exemplars” [10, 14, 15]. This distinction is based on the fact that, in models of general phenomena, the phenomenon is both instantiated in and represented by the MO. In contrast, MOs representing human diseases do not spontaneously develop the disease; rather, some features of the disease are induced by manipulating the organism.

BOX 2 **Why model organisms can be considered as models representing human patients.**

One might consider it naive to think that an animal could represent a human patient. Consider Huntington's disease, which results from a mutation in the gene coding for the huntingtin protein. This mutation leads to neuronal loss in a region of the brain called the striatum, resulting in motor symptoms. By introducing the mutation into the mouse genome, a model is established, with the aim of representing the human patient. Clearly, stating that a model represents its target does not imply equivalence between the model and the target. Rather, MOs function as surrogate models when a more direct investigation of the target is not feasible. In this view, MOs serve as substitutes for another organism (e.g., the human patient). However, results obtained from a MO can be generalized beyond the organism studied only if it adequately represents the target. Thus, rather than a simplistic, naive identification between the model and its target, stating that a model represents a target means that the knowledge gained from studying the model can be projected onto the target. Yet some scientists remain skeptical of calling an organism (or its parts) a “model of.” Orthotopic models, in which human tumor cells are grafted into the corresponding organ of a mouse, illustrate this stance [16]. The objective is to recapitulate the natural environment, given that tumor growth and metastasis processes involve complex interactions between cells of different types, the extracellular matrix, and the vasculature. A mouse entails more variables that regulate tumor growth than an empty petri dish; making it an adequate system for studying tumors. However, one might be reluctant to agree with the idea that a mouse grafted with tumor cells represents a human patient. One would rather state that an orthotopic model is defined as setting up the conditions that include all relevant variables in order to conduct better experiments. This operational definition avoids the use of the term “model,” but it must be acknowledged that the results obtained from this experiment will contribute to the general knowledge on cancer and the development of therapeutic strategies. In fact, the results obtained are projected beyond the organism, and nothing more is expressed by the statement: “this model represents this target”. Moreover, the notion of representational target is helpful, for evaluating how knowledge derived from MOs might extend beyond the specific organisms studied, as it invites reflection on their validity through concepts from the philosophy of science.

A MO can represent only one aspect of the target, and different MOs can be used to represent the same target, both strategies contributing to the representational fidelity. Most models used to study human diseases do not capture every aspect of a patient, and many MOs represent only one symptom or a subset of the biological processes involved. Conceiving MOs as models of specified components of the target is a strategy that strengthens the justification that a given organism is a good model for a target. For example, it is difficult to claim that a mouse fully represents patients, but it is more justifiable to consider a reasonable level of similarity by using a mutant mouse to study cellular alterations, associated molecular mechanisms, and specific behavioral symptoms [17]. In parallel, using multiple MOs to explore a single target can clarify which principles and mechanisms are general and which are species‐specific. It is noteworthy that, while building models is guided by the aim of similarity, seeking differences can also help to distinguish general from specific mechanisms or functions. Knowledge thus grows from the confrontation of results from several MOs [10, 18]. Hence, non‐traditional model organisms provide novel perspectives on biological questions, and allow us to assess biological principles in their full complexity [19, 20, 21, 22]. The same logic applies to human disease: multiple MOs each capture different facets of a pathology and collectively advance preclinical research [23].

Finally, to be exhaustive, it should be noted that there are cases in which MOs do not play a representational role (Box 3).

BOX 3 **Non representative use of model organisms.**

Despite general agreement that MOs frequently serve a representational role, debates persist in philosophy of science over whether representation is always involved ([24]; [25]; [26]; [27]; [28]). Two cases often lie outside this representational framework. First, organisms are studied in what has been called “preparative experimentation” [29]. Through studies done on MOs, practical knowledge, protocols, and tools are developed (e.g., antibodies, RNA interference techniques from *C. elegans* , or the cre‐lox system from *E. coli* ). This expertise—for example, in rearing conditions or genetic engineering—shapes how experiments are conducted and interpreted. In some laboratories, developing and validating such tools is the sole purpose of MO‐based research, rather than modeling an external target. Second, research may be driven with the aim of reproducing a property held by the organism. For example, in the field of biomimetics, spider silk's elasticity and strength motivate research into replicating it, without necessarily representing the web‐building process. Another case is that of naked mole‐rats, which are resistant to cancer. They are studied to gain insight into how this resistance might be harnessed, rather than to represent a disease. Thus, while MOs most frequently are representations of a target, they also serve as practical instruments for tool development or as sources of unique properties to be emulated.

#### 2.2.2 The Representational Scope of MOs

If the target of a MO is a particular biological process (for instance, apoptosis in *C. elegans* ), defining that target leaves open the question of whether the results apply only to other *C. elegans* individuals, or can be generalized to other worm species, or even to insects or mammals. The extent to which findings can be generalized beyond the organism under study is the “representational scope,” or “external validity.” In other words, it is the degree to which a specific result can be extended to broader contexts or populations. A key feature of MO research is that validity typically draws on an evolutionary background [29]. Known phylogenetic links can justify attempts to extrapolate findings, but they do not guarantee that a biological process will actually generalize. Indeed, the scope cannot simply be anticipated based on the biological process or function, and gene homology (Box 4). There may be differences between species that prevent the generalization of the biological processes described despite the existence of well‐established phylogenetic links. This evolutionary background is not intended to erase these differences, but rather, to make the generalization a priori plausible. Depending on the research objectives, the scope of a given MO can be narrow—applying only to other individuals of the same species, for example the cellular mechanisms of color change in camouflaging cuttlefish [30]; or broader, as seen in studies on aging or mitochondrial biology in *C. elegans* [31, 32] as well as in studies of human genetic diseases with non‐mammalian models [33]. For studies where the scope is limited to one species of an organism, two scenarios can be distinguished. Either the target exists only in one species, in which case the scope is limited to individuals of that species; or the target has specific features that limit the scope to one species but the results of the study provide knowledge about a more general target beyond this limited scope. For instance, the singing mouse ( *Scotinomys teguina* ) serves as a model to study the neural mechanisms of social vocal interaction, but its specific vocal behavior limits the scope to this species [34]. Nonetheless, the integration of results from multiple models—singing mice, zebra finches, vervet monkeys, and humans—helps to build a conceptual framework for vocal interaction [35]. In other words, although the specific features of the target limit the scope, the results can be transferred, not to be generalized to another species, but to contribute to a general understanding. This is because the target is not represented by any single MO, but is built from knowledge gained from several MOs, despite their limited scope. Comparing the physiology and cellular mechanisms that sustain similar functions across a diversity of MOs helps to clarify general functional principles [18].

BOX 4 **Representational scope across phylogenetic distance: a priori assumption, a posteriori justification.**

Generalization, or external validity, is not solely determined by the choice of a particular MO. Each target has a scope that can range from narrow to broad. This range can be estimated using a priori assumptions that take into account phylogenetic distance and the nature of the target phenomenon or mechanism. Indeed, one would expect exocytosis to be a low‐level, well‐conserved mechanism with a wide scope, while a high‐level phenomenon such as food intake‐related obesity would have a narrow scope, limiting its study to mammalian models. However, neither phylogenetic distance nor the low/high‐level distinction provides necessary or sufficient conditions. On the one hand, exocytosis mechanisms share strong similarities between unicellular eukaryotes and human neuronal synapses [36], but mouse and human synapses differ in the content of key proteins [37]. On the other hand, knockdown of orthologous genes related to obesity in humans prevents fat accumulation and improved life span in *C. elegans* that overconsume fructose [38]. Thus, the low/high‐level distinction in terms of phylogenetic distance is not a sufficiently reliable basis for determining the scope of the results. Similarly, a simple assumption of extrapolation based on gene conservation alone may be misleading. For example, a gene encoding an important synaptic protein found in the metazoan relative *Salpingoeca rosetta* retains conserved protein domains, but is localized to the nucleus and performs unrelated functions [39]. Thus, highly conserved genes encoding similar proteins may underlie strikingly different mechanisms and functions. A better estimate would come from reporting the conservation of several genes in combination with associated mechanisms at the protein level. These considerations lead to the conclusion that scope is better estimated a posteriori and by comparing results obtained from different MOs. Extrapolation of results is evaluated by comparing and ranking them according to their source (from *C. elegans* to primates, or more reduced models, such as cell lines) and experimental conditions. Thus, the scope is assessed using a holistic strategy of multiple comparisons, and is constantly interrogated and re‐evaluated [40].

#### 2.2.3 MOs Are Exemplars

Another unique feature of MOs is that they are actual specimens or exemplars of the biological world. This implies that, unlike computational or physical models which may omit unknown yet crucial parameters, MOs are specimens the model contains all parameters, known and unknown. This provides essential grounds for validity. Note that this is essential but not sufficient: if certain variables are present but overlooked, they will not factor into the interpretation of results, even if they play a causal role. Nevertheless, since MOs are exemplars and not reduced models, incremental studies can gradually uncover these hidden variables. In that sense, MOs are both idealized models and exemplars.

#### 2.2.4 MOs Are Artifacts

MOs are not only exemplars of the biological world; they are also artifacts. Philosophers define an artifact as an object or tool created or modified by humans for a specific function. This should not be confused with an “artifact” present in an experimental measurement. For instance, the widely used C57BL/6J mouse strain is an artifact because inbred, isogenic animals do not occur in nature. Similarly, genetically modified zebrafish, mice, or primates are artifacts.

#### 2.2.5 MOs Are Plastic

MOs also differ from other models because they exhibit homeostasis and plasticity. Homeostasis is the principle that encompasses all mechanisms by which organisms constantly maintain themselves in a steady‐state. Plasticity implies that an experimental intervention induces systemic adaptation at the genetic, cellular, or physiological level. These properties are both highly relevant when considering organisms as models. For example, in genetically modified organisms, genetic compensation can occur. This means that the phenotype results not only from the edited gene, but also from changes in the expression of other genes responding to that single manipulation [41]. Thus, a MO is an artifact that alters itself, often in ways the scientist may not fully anticipate. For example, specifically targeted manipulation of neurons also induce systemic effects at the level of the brain [42]. This observation contradicts the above‐mentioned idea that MOs are inherently valid simply because they are exemplars: when we do not know which variables are affected by manipulation, generalizing findings becomes more difficult. Phenotypic plasticity—the capacity of a single genotype to produce different phenotypes under varying environmental conditions [43, 44]—is equally significant for defining and evaluating MOs. For instance, a rodent kept in a laboratory cage is not equivalent to one living in the wild. Indeed, it is well established that housing conditions can greatly affect organisms: enriched environments alter brain structure, connectivity, and gene expression [45, 46], and voluntary exercise (e.g., running wheel) leads to physiological adaptations in mouse models of muscular dystrophy [47]. Consequently, housing and breeding conditions are tightly controlled and standardized, making MOs as much artifacts as exemplars of natural species [12, 48, 49]. Thus, the plasticity of living organisms constrains the validity and the generalization of results.

#### 2.2.6 MOs Are Experimental Systems

The abovementioned features imply that MOs should be considered within the experimental conditions studied, as different experimental setups will frame the phenomena produced by the organisms. For example, to understand how the sensory cortex integrates information about the environment, the neural activity of the rat somatosensory cortex was recorded while its whiskers were mechanically stimulated. Initial studies were performed on anesthetized rats. Then recordings were made on head‐fixed awake animals. Finally, further studies were performed to correlate recorded cortical activity with whisker touch in freely moving animals. Not only was the brain activity of anesthetized and awake animals different [50], but a striking difference was also found between passive (i.e., head‐fixed) and active (i.e., freely moving) tactile stimulation [51]. Hence, what is represented by a MO cannot be considered independent of the experimental conditions or the chosen measured parameters. In the philosophy of experimentation, it has been described as “experiments having a life of their own”: the settings of an experiment establish the conditions under which a phenomenon occurs and shape it [52, 53], and the relationship between the model and the tools that allow its manipulation and development is constitutive [54, 55]. MOs can be described as experimental systems being composed of the organism itself, the experimental protocols, and the conceptual framework mobilized to justify experimental choices (e.g., definition of the target, concepts used to interpret results) [56] (Figure 1).

#### 2.2.7 MOs as a Way to Do Science

Finally, it is crucial to note that MOs undergo a process of standardization at multiple levels: the organism itself, the housing conditions, the experimental protocols, and the tools used for measurement and manipulation. This process is shaped by the scientific community and framed by infrastructures [12, 57]. These collaborative efforts of these communities produce key resources, such as reference genomes and neural connectomes, which are shared via platforms like WormBase and FlyWire [58, 59]. The coordination of a community improves and stabilizes the way research is conducted, as seen for example with the zebrafish model [60]. It also enables models to be established, as in the notable case of the marmoset, which became a MO following the initiative of Japanese neuroscientists [61]. Consequently, a final defining feature of MOs is that they depend on the availability of infrastructure, community support, and shared tacit knowledge for continued use as models.

## 3 How to Establish the Validity of a Model Organism? Existing Proposals

### 3.1 Phylogeny, Reductive Account, and Empirism

It has been proposed that generalizing results in the life and biomedical sciences often relies on qualitative comparisons and background assumptions, contrasting with the more formal justifications grounded in universal laws typically sought in physics and chemistry [62]. According to this view, the external validity of MOs rests on two primary arguments. First, phylogeny serves as a background assumption, positing that organisms share evolutionarily conserved features [25]. In this context, the term “assumption” means that the validity of phylogeny‐based generalizations is accepted as true rather than being formally proven—without, however, calling evolutionary theory itself into question. Second, higher‐level phenomena (such as diseases or behaviors) are studied at the cellular and molecular levels, where transposition from one organism to another may be more straightforward [63]. This suggests that the mechanisms or modules of phenomena identified in simpler MOs serve as building blocks, which increase in number and complexity in higher organisms. This perspective is exemplified by the proposed relevance of the lamprey for understanding motor control in mammals [64]. External validity would thus be supported by a hierarchical structure of biological explanations. According to this framework, fundamental phenomena arise from conserved low‐level mechanisms, and more complex phenomena build upon them [63, 65]. However, justifying external validity in MOs through phylogenetic assumptions and the notion that complex phenomena can be reduced to simpler, lower‐level mechanisms is a matter of philosophical debate (Box 4). The reductive accounts employed [66] and the debatable validity of inferences used to support generalizations [67, 68] have led to the view that the justification of external validity remains largely empirical [40, 63].

### 3.2 Willner's Three Criteria

Another approach to assessing external validity in MOs can be found in studies on animal models of depression. In this context, Willner proposed three criteria: face validity, predictive validity, and construct validity [69]. While these criteria have gained traction among researchers evaluating biomedical models of various psychiatric disorders, they also pose certain difficulties. Face validity refers to the similarity between phenomena observed in the MO and those seen in human patients. However, symptoms of depression in humans are modeled using behavioral tests on rodents that involve behaviors not observed in humans. These tests are considered valid mainly because existing antidepressants reverse these behaviors in rodents. Consequently, since no direct parallel exists, it is difficult to view face validity as a straightforward measure of external validity. Predictive validity requires pharmacological treatments to have similar effects in the model and in human patients, but this criterion can only be confirmed a posteriori. Finally, construct validity requires that the modeled disease features in animals are demonstrably homologous (both empirically and theoretically) and can be interpreted unambiguously. However, this criterion is polysemic, leading to diverse interpretations [70]. Willner's three criteria nevertheless positively impacted biomedical research. They have stimulated reflection on the validity of rodent models of psychiatric disorders [15, 71, 72, 73] and led to further conceptual developments [70]. However, it remains difficult to derive a universal, performative framework for all types of MOs based on these criteria.

The example of psychiatric disorders also highlights a general point about MOs: evaluating whether a target is well represented presupposes that the target itself is well defined. When a phenomenon is poorly defined or remains a topic of debate, additional conceptual work is required before attempting to assess a model's validity. The challenge of defining psychiatric disorders illustrates this issue, complicating model validation [74, 75, 76].

### 3.3 Analogical Reasoning

A third approach stems from the representational account of MOs, and suggests that validating a model's representation of its target depends fundamentally on analogical reasoning. According to this view, a model is considered valid if it demonstrates relevant similarities to its target. However, analogical justifications share many philosophical challenges with inductive reasoning, making them difficult to establish conclusively. In its simplest form, analogical reasoning can be described as follows. Consider two domains, a source domain and a target domain, each of which encompasses certain objects, properties, or functions. If these domains share some similarities (i.e., have common features) and a new feature is observed in the source domain, one may infer that the target domain possesses this feature as well. Therefore, analogical reasoning is an inference by which a feature of a source domain is attributed to a target domain, thereby yielding new knowledge about the target domain. Various approaches have been proposed to justify (i.e., prove to be true) analogical inferences [77] (For clear and concise explanations on analogical reasoning applied to animal models, see [23, 56]).

One influential perspective is the structure‐mapping theory, which argues that analogies are better grounded in relations rather than in isolated features [78]. Under this structuralist view, extrapolation is not justified by listing discrete similarities, but rather by mapping how relations among features in the source domain correspond to those in the target domain. Shelley applies structure‐mapping theory to show that valid analogies between a MO and its target depend on the identification of systematic correspondences in their underlying relationships, rather than on superficial similarities [79]. Mapping these deeper relational structures across different biological levels (molecular, physiological, behavioral) strengthens the model's capacity to generate reliable inferences about its target. Shelley's proposal demonstrates that extrapolating findings from a MO to its target becomes more justifiable when they share a well‐structured network of relations, thereby reinforcing the model's overall validity.

Steel's comparative process tracing (CPT) offers another proposition for justifying extrapolation by identifying and comparing the causal mechanisms of a model and its target. As Guala summarizes, the idea is that “an inference from laboratory conditions to a non‐laboratory (target) system is validated by cross‐checking the crucial nodes of the relevant causal mechanisms” [80]. Unlike purely analogical reasoning, CPT involves three steps: (1) experimentally determining the causal pathway in the model, (2) identifying points where the mechanisms in the target are likely to diverge, and (3) assessing whether these differences are significant enough to prevent extrapolation. Rather than requiring complete mechanistic identity, CPT relies on relative similarity at critical stages, making it applicable to both biological and social sciences. For instance, when studying the carcinogenicity of aflatoxin B1, CPT revealed that, although mice and rats share broad metabolic similarities, mice possess an additional detoxification mechanism that is absent in humans. This finding justified the extrapolation from rats, but not mice, to humans when assessing aflatoxin B1's carcinogenic potential [80, 81, 82]. Beyond the argument that low‐level mechanisms are conserved through evolution—thus justifying extrapolation from MOs [63]—the identification of mechanisms is often invoked as “mechanism‐based evidence.” Discovering these mechanisms allows researchers to move from correlational to causal claims, strengthening external validity [83]. When a mechanism cannot be demonstrated directly in the target (e.g., when human experiments are unfeasible), evidence becomes robust if the same mechanism is observed in multiple other species [84]. However, in biomedical research, which seeks to extrapolate findings to humans, mechanistic extrapolation is often difficult to justify because it requires tracing causal links. Yet direct human data are often unavailable, and the relevant mechanisms remain unknown [85]. Moreover, the practical goal of biomedical studies is often prediction rather than explanation. For these two reasons, researchers may rely instead on another type of inference: statistical extrapolation based on difference‐making evidence. This approach justifies predictions when past model‐to‐target predictions have been successful (e.g., if drug X has similar effects in mice and humans, one might infer that another related drug that is effective in mice will also be effective in humans) [86].

## 4 A New Framework for Evaluating MO's Validity

**FIGURE 2:**
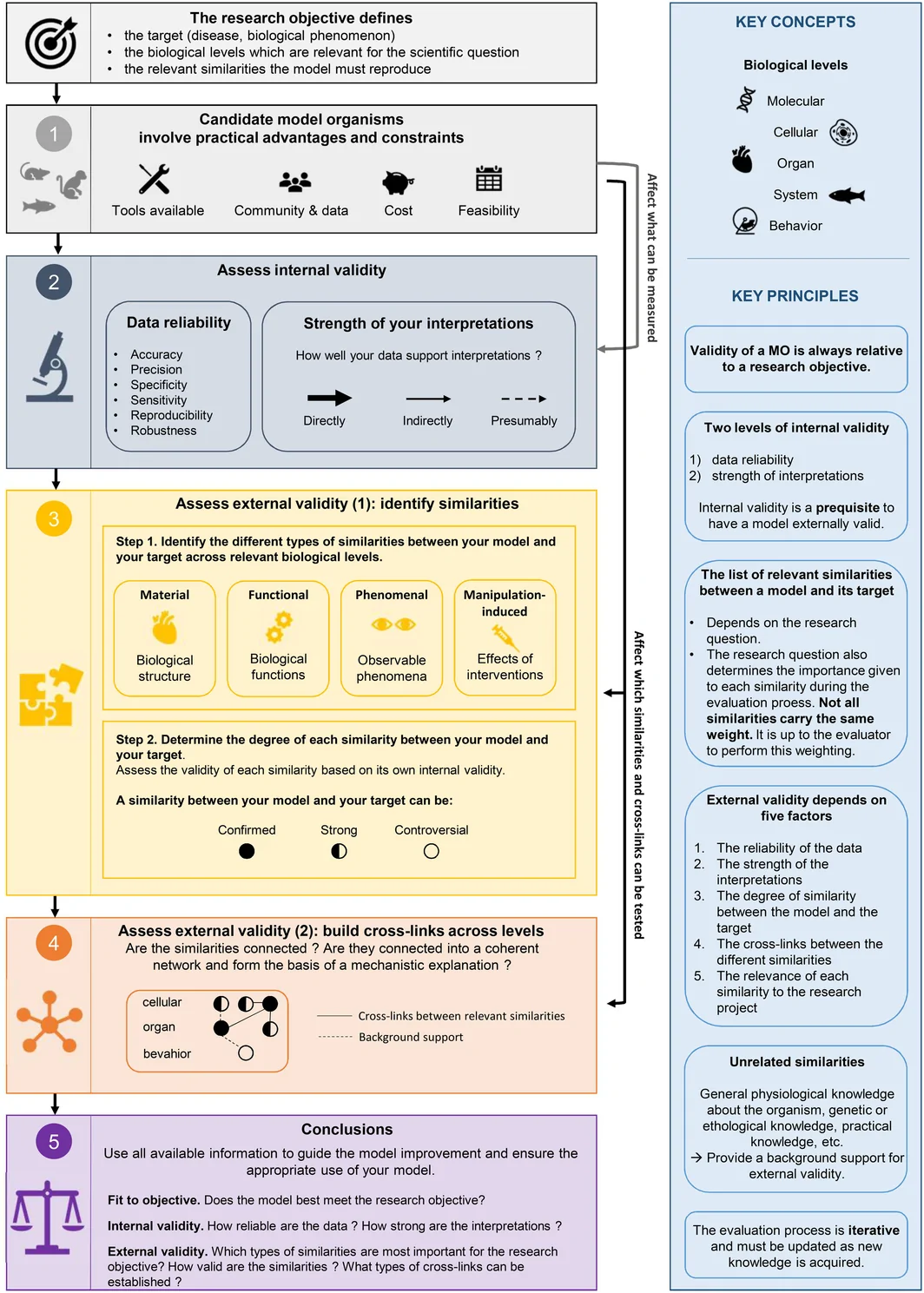
Workflow for evaluating a model organism: The evaluation of model organisms is structured as a stepwise workflow. (1) First, the research objective is defined by specifying the target and the relevant biological levels. Candidate organisms are then identified while making explicit practical constraints such as available tools, feasibility, cost, and community resources. (2) Second, internal validity is assessed by evaluating both data reliability (accuracy, precision, sensitivity, specificity, reproducibility, robustness) and the strength of the interpretations. (3) External validity is then approached by identifying similarities between the model and the target, classified into four types: material similarities, functional similarities, similarity of phenomena, and similarity induced by manipulation. Each similarity is assigned a degree according to the internal validity and the correspondence between the results obtained in the model and the target. (4) These similarities are then integrated through cross‐links, which reflect the coherence of the underlying explanatory structure and may correspond to mechanistic relationships. The cross‐link network is also supported by background knowledge. (5) Finally, several models can be compared by integrating their respective internal validity, the number and degree of their similarities, and the strength of their cross‐links. This comparative assessment supports the context‐dependent choice of the most appropriate model organism by making the trade‐offs between competing options explicit. The process is iterative because new data may modify the evaluation of similarities and their relevance to the research objective.

**FIGURE 3:**
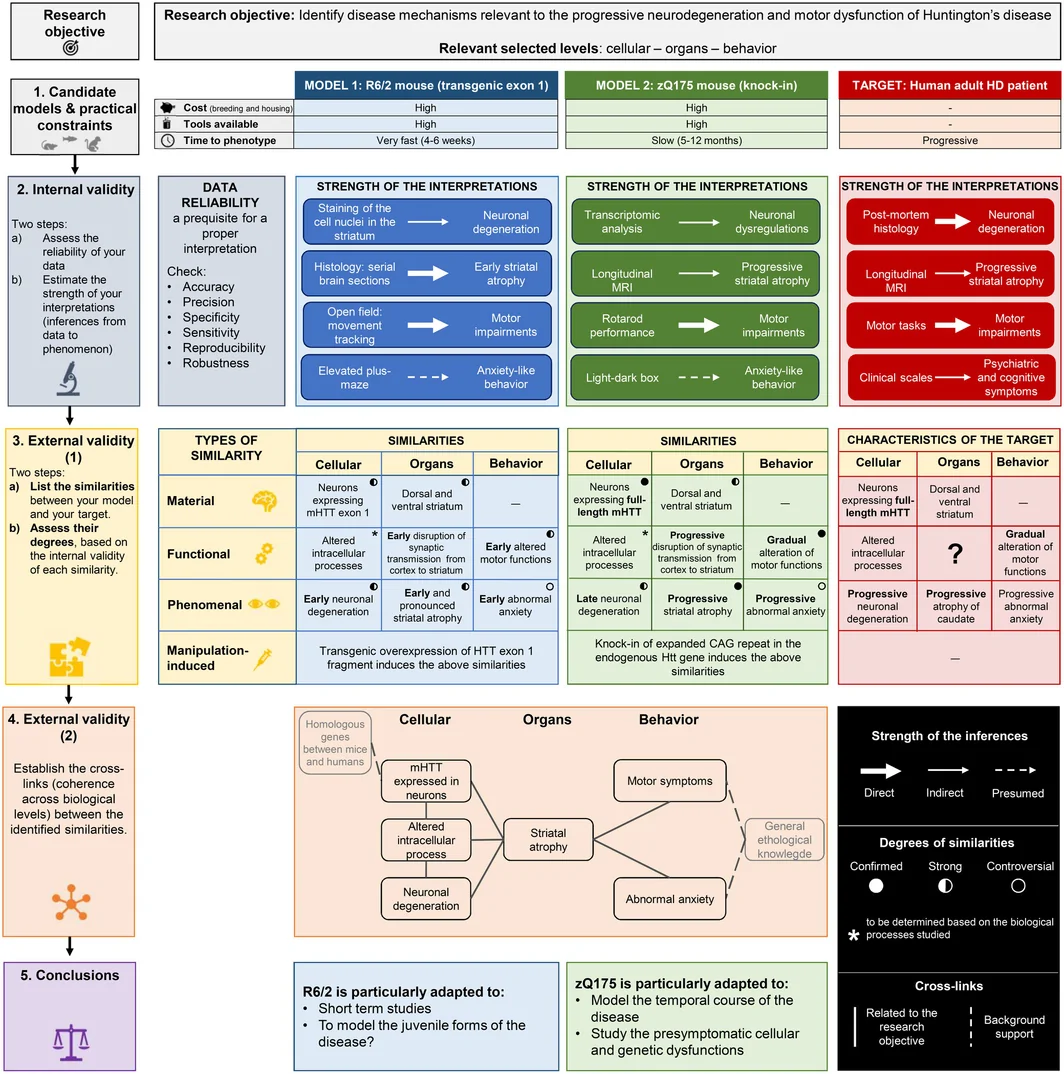
Exemplified application of the framework to evaluate model organisms for the study of a human disease. The framework is applied to the R6/2 and zQ175 mouse models, using adult patients with Huntington's disease (HD) as the target. The experimental procedures shown are illustrative examples of methods that may be used in these models and do not constitute an exhaustive list. Arrow styles indicate whether inferences from data to phenomena are direct, indirect, or presumed, whereas circles indicate whether similarities are confirmed, strong, or controversial. Indirect measurements may support a confirmed similarity when the inferred phenomena are concordant and supported by established background knowledge, but only a weaker similarity when concordance or background support is limited. Cross‐links represent either relationships relevant to the research objective or relationships supported by background knowledge. An asterisk (*) indicates that the degree of similarity must be determined according to the biological processes under consideration. A question mark (?) indicates that no evidence is currently available for the corresponding characteristic in the target; it does not indicate evidence of absence. mHTT denotes mutant huntingtin (HTT). In zQ175 mice, overt neuronal death is absent; however, progressive cellular dysregulations provide access to a temporal aspect of the disease that is also observed in humans. Consequently, R6/2 mice are particularly suited to short‐term studies and potentially to modeling juvenile forms of HD, whereas zQ175 mice are better suited to studying the temporal course of the disease and presymptomatic cellular and genetic dysfunctions.

**FIGURE 4:**
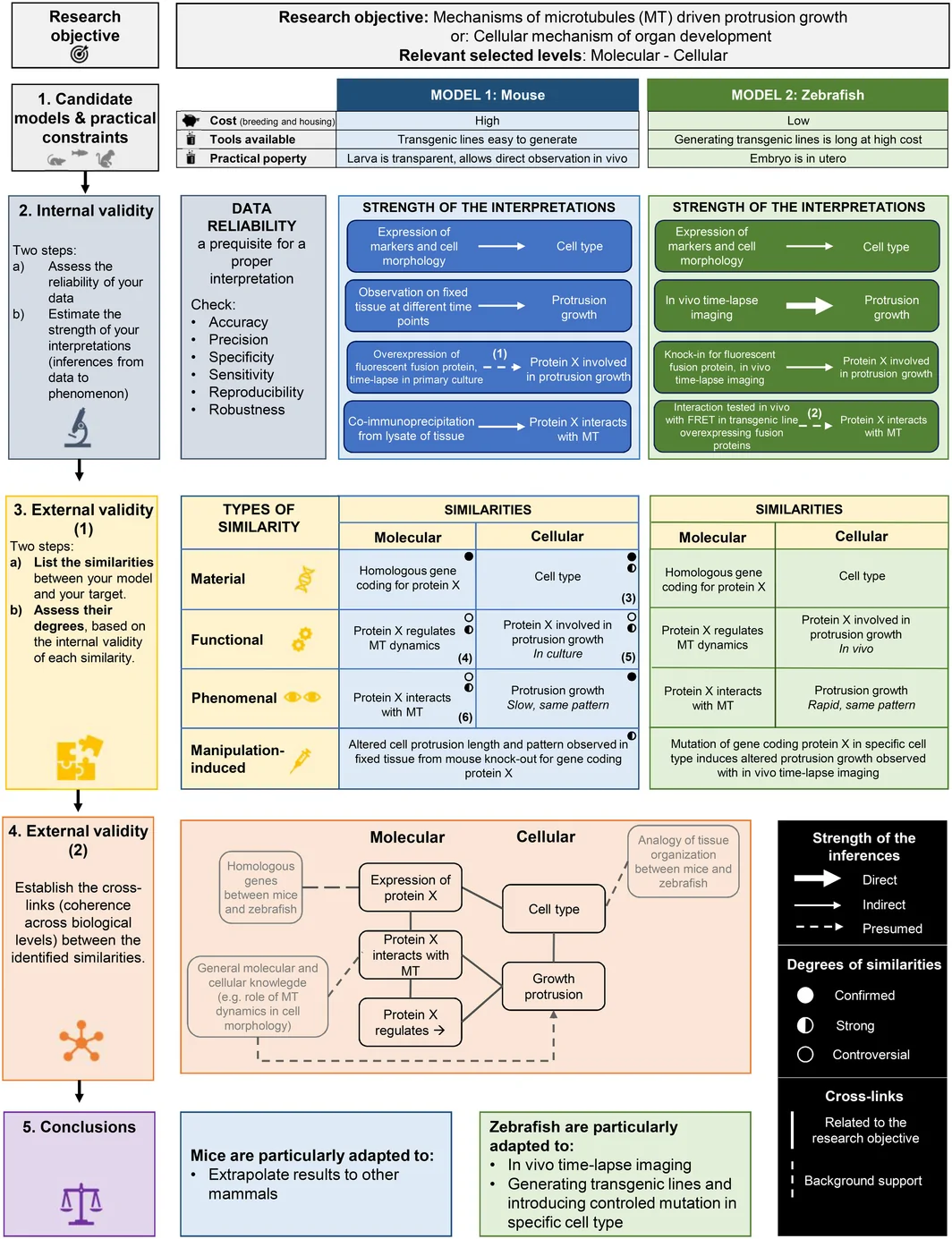
Application of the framework to evaluate model organisms for the study of either molecular mechanisms of cellular phenomenon, or molecular and cellular mechanisms underlying organ development the framework is applied to MOs whose representational target is a cellular phenomenon. Two different research objectives are defined, to illustrate how it frames the evaluation. Drawing on studies of the role of Fidgetin in microtubule dynamics and motor‐neuron axon growth, the figure presents a generalized example. (1) The role of protein X in protrusion growth is presumed in the mouse model because the experiment is conducted in primary cell culture, as the phenomenon cannot be observed directly in utero. (2) The interaction between protein X and microtubules is presumed in the zebrafish model because detecting protein interactions using FRET requires overexpression, which may produce false‐positive results. (3) The similarity is confirmed if concordant results are obtained in other species; otherwise, it is classified as strong. (4) This functional similarity is presumed because it is based on an assumption derived from the results presented in the table, but it could be classified as strong if supported by new results. (5) Because the inference in the mouse model is presumed—the results were obtained from primary cell culture rather than in vivo—the similarity may nevertheless be classified as strong if the research objective concerns molecular mechanisms alone, but as controversial if it concerns the mechanisms underlying organ development. In the latter case, observing an in vivo phenotype following manipulation of gene expression, such as a knockout in mice or a mutation in zebrafish, supports reclassifying the similarity from controversial to strong. (6) Because the inference in the zebrafish model is presumed—testing protein interactions under overexpression conditions has limited internal validity—the similarity may nevertheless be classified as strong if the research objective concerns molecular mechanisms alone, given the co‐immunoprecipitation results obtained in mice. However, it is classified as controversial if the research objective concerns the mechanisms underlying organ development. In the latter case, applying the method used in the mouse model to zebrafish could lead to a reclassification of the similarity from controversial to strong.

In the following sections, a new framework is proposed that allows us to evaluate the validity of any organism model in all aspects of the research process. This framework is organized as a five‐step workflow: (1) making the practical constraints (or values) that guide the choice of the MO explicit; (2) assessing internal validity based on data reliability and interpretations; (3) assessing external validity based on identifying different types of similarities between the model and its target; (4) integrating these similarities across biological levels through cross‐links; and (5) synthesizing these elements to guide model use, comparison, and refinement. The framework is summarized in Figure 2 and illustrated with two examples in Figures 3 and 4.

From the outset, it is important to note that two factors shape the evaluation. First, phylogenetic relationships provide a background that may guide expectations of similarity without guaranteeing them (Box 4). Second, and more importantly, the entire evaluation must be conducted in light of the research objective, as this determines which similarities and which levels are relevant. Therefore, the same model may be appropriate for one scientific question but not for another (Box 5).

BOX 5 **Summary of the framework for evaluation of the validity of model organisms.**

This framework makes it possible to evaluate the validity of any model organism, as the target represented by the model can be either a human patient or a general biological process or function. Validity refers to the extent to which knowledge derived from studies conducted using a model organism (MO) can justifiably be generalized to the target. First, the framework allows to assess internal validity. The reliability of the data is evaluated with defined criteria for measurements, and the validity of the results is estimated by determining whether interpreting the data as evidence of a specific phenomenon is justified. Then, external validity is assessed through several steps, using multiple types of similarities that are both descriptive (i.e. reflecting how researchers actually work), and normative (i.e. representing epistemic criteria for evidence‐based reasoning). Together, these four types of similarities capture both mechanistic and predictive evidence. Each type of similarity provides some evidence with a certain degree, depending on the internal validity of the results. This notion of degree allows to articulate internal and external validity. Each identified similarity is positioned at a particular level of investigation (molecular, cellular, physiological, etc.). The level itself is not indicative of validity (i.e. a similarity at a molecular level is not intrinsically more valid than a similarity at a behavioral level), but mapping similarities onto levels permits a structural analysis of external validity. In the final step of the evaluation process, cross‐links are built between similarities across levels to provide an overall picture of the external validity. A global assessment of how well the model represents the target, and whether the results can be generalized, emerges from considering the number of similarities at each level, their degree, and how they are linked or interrelated to provide explanatory evidence. Two further considerations must be taken into account: phylogenetic distance and the research objectives. Phylogenetic distance is a background assumption, but it does not support validity along a linear scale. Instead, validity must be assessed by considering each similarity in relation to the particular organism involved. Validity must also be assessed in light of the research objectives, which guide the selection of relevant similarities. Thus, phylogenetic distance and research objectives both shape the evaluative framework.

### 4.1 Step 1. Making Values Explicit in the Choice of the Organism

Values, as named in philosophy of science, shape and constrain the choice and conduct of research projects [87, 88]. In particular, non‐epistemic values, such as cost, reproductive rate, community support, and ethical considerations, influence the choice of a MO (for an overview on values in the choice of MOs, see [89]). Given the influence of such practical considerations on the production of knowledge, it is a prerequisite for any evaluation to make explicit the practical factors involved in choosing one organism over another, and to determine the adequacy of the organism for assessing a particular research question (Figure 4).

First, the choice of the organism depends on whether the biological process of interest can be observed or manipulated to reveal its underlying mechanisms. Valid results are obtained only if an organism allows accurate measurements or specific interventions. Recent tools can however allow new organisms to emerge as models [20, 90]. Next‐generation sequencing and the advances of proteomics analysis allow us to propose a diversification of organisms as models [19, 33]. Another example is cnidarians, such as jellyfish and hydra, that represent an interesting target: a simple nervous system that controls coordinated behaviors. These animals have become a model for elucidating the fundamental principles of neural systems since gene editing has been applied to them for observation with gene‐encoded calcium sensors and intervention with gene‐encoded optogenetic actuators [91]. Thus, when selecting an organism, it is important to ensure that the experimental tools can provide accurate data and allow precise interventions to support generalization of the results.

Second, tensions often arise between these practical imperatives and epistemic goals [92], which may in turn affect external validity. For example, the R6/2 mouse model of Huntington's disease, a genetic disease with progressive degeneration of a brain region that controls motor coordination and locomotion, develops symptoms rapidly, enabling faster results but potentially compromising external validity due to its divergence from the slow progression seen in human patients (Figure 3).

In some cases, however, practical and epistemic concerns converge. For example, the anemonefish was adopted as a model because the combination of experimental setups and the properties of that fish allowed conducting an Eco‐Evo‐Devo approach (Ecology combined with the Eco‐Devo approach) [93]. In general, if a model is widely adopted by multiple research teams because of its practical advantages, extensive knowledge about it will likely accumulate. Thus, it is possible for a practical consideration to function as an epistemic criterion [94, 95].

### 4.2 Step 2. Estimating Internal Validity: Reliability of the Measured Data and Strength of the Interpretations

Internal validity concerns the reliability of the data produced by an experiment and the justification of the interpretations of these data. In biology, as in other experimental sciences, the experimental process progresses from the collection of data to their interpretation into phenomena. This distinction is especially important because phenomena are most often inferred from data rather than directly observed. In other words, an inference is required to conclude that data are indicative of a phenomenon [96]. For example, stress is a physiological state that may be assessed by measuring the blood level of interleukin‐6 (IL‐6), rather than directly observed. Thus, internal validity involves two levels of inference: the reliability of the measurement itself (e.g., the accurate measurement of IL‐6 level) and the inference that the data indeed indicate a phenomenon (e.g., stress). Therefore, the reliability of the data and the validity of the results must be justified through distinct criteria in the evaluation process.

#### 4.2.1 Data Reliability

Data can be defined as the outcomes of measurements, or as quantified variables. In the philosophy of measurement, four criteria are generally used to estimate the data reliability: accuracy, precision, specificity, and sensitivity (Table 1) [97]. While accuracy is advisable in principle, the interest usually lies in estimating differences between experimental conditions rather than in estimating the absolute real value. Similarly, the required precision depends on what is expected to be observed and the question posed. Sensitivity refers to the detection threshold and signal saturation. If the true value to be measured is lower than the detection threshold, the outcome of the measurement will be a false negative. Conversely, if the value to be measured is above the detection range of the measurement apparatus, the outcome of the measurement will be a fixed number that does not correspond to the true value. Increasing the sensitivity of measuring devices has enabled research on MOs that were previously inaccessible. For example, the Xenopus oocyte was historically used to study the cell cycle due to its ability to provide a large quantity of proteins [98]. Consequently, these criteria may also depend on the type of organism used.

**TABLE 1:** Criteria of internal validity.

Internal validity criteria	Criteria details
Data reliability	Accuracy: Measured data are close to the real value
	Precision: Measured data are close to each other when repeated
	Specificity: Measurement retrieve data only on the selected parameter
	Sensitivity: Detection threshold and signal saturation limit measurement range
	Reproducibility: Convergent data are found when the experiment is repeated
	Robustness: Convergent data are obtained with different techniques
Strength of the interpretations (inference from data to phenomenon)	Direct: Inference is based on direct measurements
	Indirect: Inference is based on indirect measurements, and valid because the relevant background knowledge is well established
	Presumed: Inference is based on indirect measurements, but relevant background knowledge is incomplete or the inference relies on assumptions.

In experimental biology, however, the reliability of data depends not only on the measurement instruments, but also on the organism being studied. This biological dimension adds a layer of complexity to the assessment of the reliability of data. Even with highly accurate and precise instruments, inter‐individual or inter‐organism variability inevitably leads to differences in values obtained from repeated measurements. This variability is not due to a lack of precision or accuracy of the instruments, but rather to the intrinsic nature of the biological systems under study. Thus, to evaluate the reliability of data, it is critical to repeat measurements on multiple samples from different individuals. This reproducibility—defined as the repetition of the same measurements on multiple individuals—allows for the validation of measurement reliability while accounting for biological variability. One factor that affects reproducibility is data dispersion between individuals, which varies by organism: it is low in *C. elegans* , but much higher in CD1 mice. To increase the reliability of data, it is also important to use independent measurements that indicate the same phenomenon [99]. In addition, the robustness of data is strengthened when the same findings can be obtained independently using different methods [100].

#### 4.2.2 Validity of Results: Strengths of Interpretations (Or Inference From Data to Phenomena)

Once we establish that the data obtained through our MOs are reliable, we can infer a phenomenon from them. However, providing specific criteria for this inference is considerably more challenging than assessing data reliability, for several reasons. First, inferences from data to phenomena can vary widely, encompassing anything from inferring the death of a cell type to determining the cognitive state of a rat. Second, these inferences are built upon prior experiments and accumulated knowledge, both of which remain open to revision. Finally, it is not only the epistemic background that is crucial for evaluating these inferences but also the entire experimental context. The dependence of these inferences on the epistemic and experimental background frequently gives rise to disagreements among researchers.

Nonetheless, the strength of the inference from data to phenomenon can be evaluated by distinguishing three types of inference: (i) direct, (ii) indirect and (iii) presumed (Table 1). (i) A direct inference is based on direct measurement; therefore, the inferential distance between the data and the phenomenon is minimal. For example, in mouse models of Huntington's disease, the atrophy of a brain region, the striatum, is assessed by directly measuring the volume of the striatum. (ii) An indirect inference is based on indirect measurements but is considered valid because the relevant background knowledge is well established. For example, IL‐6 is a valid marker of stress since the molecular and cellular mechanisms underlying its physiological role are well understood. (iii) A presumed inference is based on an indirect measurement and the relevant background knowledge is incomplete or the connection between the data and the phenomenon rests on assumptions. For instance, in the elevated plus maze behavioral test, rodents are placed on an elevated platform comprising enclosed and open arms. Assessing a mouse's anxiety level on the basis of the time it spends in the open arms relies on a risky inference.

### 4.3 Step 3. Assessing External Validity

External validity concerns whether results can be generalized beyond the model to its target. The selection of which similarities to consider depends on the specific research project, with some contributing to the validity of the MO (relevant similarity), while others have no impact (unrelated similarity).

We propose to distinguish four types of similarities between MOs or between a model and its target in order to establish external validity (Table 2). The first two types are based on the classic distinction between structure and function, which we refer to as “material similarities” and “functional similarities,” respectively. Third, we introduce the concept of “similarity induced by manipulation.” Finally, there is a fourth type of similarity that does not fit into any of the first three categories: this is what we refer to as “similarity of phenomena.”

**TABLE 2:** Types of similarities.

Similarity types	General definition	Examples from mouse models of Huntington's disease	Examples from MOs used to understand asymmetric cell divisions
Material	A similarity in biological structures (genes, molecules, cells, organs, etc.)	The dorsal striatum is present in both mice and humans.	*Par* genes are present in both fruit flies and worms.
Function	A similarity in the biological roles performed by some structures. Those structures may or may not be materially similar.	The dorsal striatum controls movement in mice and humans.	*Par* genes are critical for asymmetric division in worm zygotes and fruit flies neuroblasts.
Induced by manipulation	An experimental manipulation produces comparable effects in the model and its target, potentially across multiple biological levels.	The introduction of a mutation in the *Htt* gene produces striatal atrophy in mice, and there is a correlation between mutation of the same gene and striatal atrophy in Huntington patients.	Loss of *Par* gene's function result in a disruption of cellular asymmetry in both worm zygote and fruit flies neuroblasts.
Phenomena	A similarity grounded in the inference of a shared phenomenon between model and target, regardless of structural, functional, or manipulated commonalities.	The dorsal striatum degenerates in both mutant mice and Huntington's patients.	There are asymmetric cell divisions in both worms and fruit flies, that follow a common sequence.

#### 4.3.1 Four Types of Similarities

##### 4.3.1.1 Material Similarity

Material similarity is evaluated by determining whether the genes, molecules, cells, or organs of interest are present in both the MO and the target. Note that behavior is excluded from these material similarities. These are similarities, not perfect identities: there are no material identities between components, only resemblances between structures. In developmental biology, a simple example of material similarity between worms and fruit flies is the presence of homologous *Par* genes in both organisms [101]. Another example is the presence of the dorsal striatum—the brain region that degenerates in Huntington's disease—in the brain of both mice and humans.

##### 4.3.1.2 Functional Similarity

Often, a resemblance between biological structures indicates a functional resemblance as well. To illustrate, in developmental biology, *Par* genes are critical for establishing embryonic polarity during asymmetric cell division in *C. elegans* , and in *Drosophila* these genes play a comparable role in regulating neuroblast polarity during development [101]. Similarly, the dorsal striatum regulates movement in both mice and humans.

Nevertheless, functional similarity can exist independently of material similarity between the model and its target, allowing for multiple realizations. To illustrate this point, consider the visual system of amphibians, which can be studied because it is simpler and more accessible than those of other animals. Amphibian visual systems perform the same functions as human visual systems, yet in amphibians, these functions are primarily managed by the tectum, whereas in humans, they are mainly handled by the cortex.

##### 4.3.1.3 Similarity Induced by Manipulation

A third type of similarity is manipulation‐induced similarity. The evaluation of this type of similarity involves the assessment of whether manipulating A results in a change in B, in both the model and the target species, or across all MOs used to study a given biological phenomenon. For instance, loss‐of‐function mutations in *Par‐6* or *Par‐3* genes result in a disruption of cellular asymmetry in both *C. elegans* zygotes and *Drosophila's* neuroblasts. Indeed, the loss of these genes prevents the asymmetric positioning of a number of proteins and RNAs that are important for cell fate determination [102, 103]. Another illustration is the introduction of a mutation in the *Htt* gene, which has been observed to result in striatal atrophy in animals, and the same gene mutation is correlated with striatal atrophy in patients with Huntington's disease. In contrast to the aforementioned types, this form of similarity can encompass multiple biological levels. A phenomenon or material manipulated at one level may cause a phenomenon at the same or a higher level.

##### 4.3.1.4 Similarity of Phenomena

A fourth similarity between MOs, or between MOs and their targets, is the similarity of phenomena, which can be distinguished from both material (or structure) and function. In this way, we propose a tripartite distinction to evaluate different types of similarities: a biological structure gives rise to a phenomenon, which in turn has a function. To illustrate, the heart (structure) beats (phenomenon) to distribute blood across the body (function). For example, asymmetric cell divisions have been observed in both *Drosophila* neuroblasts and *C. elegans* zygotes, and these divisions are characterized by analogous steps. First, before division, a polarity axis is established in coordination with the body axes, followed by the orientation of the mitotic spindle along that axis. Next, cell fate determinants are asymmetrically distributed, ensuring their unequal inheritance by the daughter cells, which ultimately leads to the specification of distinct cell fates [104]. Another example is the transgenic mouse lines that exhibit motor disturbances comparable to those observed in Huntington's patients. Furthermore, both the murine model and the patient with this disease exhibit degeneration of the dorsal striatum. In such cases, the comparison between the animal model and the patient is not based on an explicit structural or functional similarity of the striatum or muscles, nor is it based on experimentally induced similarity. The comparison involves other elements. In one instance, it pertains to symptomatic similarity (motor disturbances), whereas in the other, it encompasses a shared physiological alteration (striatal degeneration), and both are best defined as phenomena. It is important to note that different kinds of measurements and experimental outcomes are often used in the model and the target. Nevertheless, these distinct forms of evidence can collectively provide a basis for inferring that the same phenomenon occurs in both. For example, motor coordination and locomotor deficits are assessed using different measurements in mice (e.g., footprint analysis of walk and the time taken to fall from a rotating rod) and in humans (e.g., clinical observation of impaired balance, walking difficulties).

#### 4.3.2 Two Main Characteristics of the Different Types of Similarity

##### 4.3.2.1 Similarities Occur at Specific Biological Levels

The described similarities must be considered according to the biological levels of interest, which can range from molecules to behavior, including genes, cells, organs, and the organism as a physiological system. The need for similarity at a particular level depends on the scientific question being addressed. In some cases, similarity at a single level may be sufficient, while in others, similarity at multiple—or even all—levels must be considered. For example, an animal model of a disease such as Huntington's disease will require similarity at multiple levels, whereas the study of intracellular events may require similarity at the molecular and cellular levels only. In other words, if the scientific question involves multiple levels, it is essential to identify similarities at all relevant levels. Here, it may be tempting to prioritize molecular‐level similarities over behavioral ones, given the notion that fundamental mechanisms are more consistently preserved, thereby enhancing external validity. However, it is important to recognize that no biological level inherently guarantees validity.

##### 4.3.2.2 Similarities Have Different Degrees

Relevant similarities between a MO and its target vary in degree: some are stronger than others. The degree of similarity depends on two factors. First, it is grounded in internal validity, as the establishment of a similarity depends on the validity of observations and experiments conducted in the MO and its target. Put simply, similarity forms a bridge whose strength (its external validity) depends on the robustness of its pillars (the internal validity). Given this interdependence, similarities can be classified into three degrees: confirmed, strong, controversial (Table 3), depending on the internal validity of the results, categorized as direct, indirect, presumed (Table 1). Second, the degree of similarity also depends on the concordance between the results obtained in the MO and in its target. The following examples clarify how the proposed approach can be used to evaluate similarities.

**TABLE 3:** Degrees of similarities.

Degree of similarity (contribute to evaluate external validity)	Validity of the results (MO and human patient or two MOs) (output of internal validity evaluation see Table 1)	Concordance between the results (case to case in light of research objectives)
Confirmed	Both are direct	Good
Confirmed	Direct and indirect or both are indirect	Good
Strong	Both are direct	Some discrepancy
Strong	Direct and indirect or both are indirect	Some discrepancy
Presumed	One or both are presumed	Good

When the inference from data to phenomenon (internal validity) is direct for results obtained in both MO and human patients, or in two MOs, and the results correspond closely, the similarity can be classified as confirmed. For example, direct measurement of striatal volume reveals progressive atrophy in both zQ175 mouse models and patients with Huntington's disease. By contrast, direct measurements in another mouse model, R6/2, reveal early and rapid striatal atrophy, whereas patients exhibit a more progressive pattern. Despite the use of direct measurements, this temporal discrepancy implies that the similarity between the R6/2 mouse model and patients should be classified as strong rather than confirmed (Figure 3).

When internal validity is indirect, the similarity can be classified as either strong or confirmed. Indirect internal validity applies when the inference from data to phenomenon is well supported, even though the phenomenon is not measured directly (Table 1). For example, dysregulation of genes encoding proteins involved in cell death may be identified though RNA sequencing. Although this measure is indirect, this similarity can be classified as confirmed when the results converge across MOs and patients with Huntington's disease [105].

Finally, when the internal validity is presumed, the similarity can only be controversial. A similarity may initially appear strong—for example, both mouse models and human patients may appear to exhibit a high level of anxiety. However, if the inference from data to phenomenon has only presumed internal validity—for instance, when anxiety in mice is assessed on the basis of the time spent in the open arms of an elevated maze—the similarity does not rest on sufficiently secure grounds. Some controversial similarities may nevertheless be widely accepted by the scientific community and presented as evidence in publications. They remain, however, dependent on inferences that require further justification. If results converge across several behavioral tests and the body of knowledge supporting the inference grows, a similarity initially classified as controversial may eventually warrant classification as strong.

Three further remarks can be made. First, the four types of similarities can be classified as confirmed, strong, or controversial. Material similarities are not necessarily confirmed, and functional similarities often involve high‐level interpretations, corresponding more closely to strong or controversial similarities. Therefore, the degree of a similarity is not contingent upon its type. Second, the level at which the similarity lies provides no indication of its degree. For example, at the molecular level, a similarity induced by a manipulation using a nonspecific tool generates off‐target effects and thus has a low internal validity. In this case, the similarity will have a low degree. Conversely, at the behavioral level, a locomotion test employed in a rodent model of muscular dystrophy can yield reliable data that are correctly interpreted as a locomotor disability, resulting in a high degree of similarity. Thus, any degree of similarity can be found at any level. Third, the research objectives, which guide the entire evaluation process, should be considered when assessing the degree of similarity (Figure 4). For example, inhibiting two different proteins that regulate the actin cytoskeleton may have similar effects on protrusion growth in the same cell type in zebrafish and mouse. This similarity induced by manipulation is strong for research aimed at understanding the role of actin in cell morphology. However, it is insufficient for research aimed at elucidating the underlying molecular mechanisms.

### 4.4 Step 4. Building a Network of Similarities Between the Model and Its Target Across Biological Levels That Integrates Internal Validity

#### 4.4.1 Cross‐Links Between Similarities Support External Validity

The evaluation of MOs using these categories of resemblances raises several issues that are difficult to avoid. We highlight the three most significant ones. (i) With regard to material similarity, it is possible for similar structures to give rise to different phenomena and fulfill different functions. In this context, material similarity may not truly strengthen a model. (ii) With regard to functional similarity, the same functions can be realized by different structures and/or biological phenomena. Thus, depending on the research question, to study a function may require models that exhibit material and/or phenomenal differences, rather than similarities. (iii) This also pertains to phenomena. Similar phenomena may manifest in different structural forms and may serve different functions.

The three identified issues suggest that a model's external validity may be significantly limited if it exhibits only one of these three types of similarity in isolation. Consequently, the external validity of the model should be considered based on the cross‐links that can be established between the different types of similarities. Moreover, the model's validity is strengthened when similarities appear across multiple levels, and cross‐links allow integration of these similarities coherently while guided by the research objectives. Indeed, as already argued, the validity of a model's representation of a target is grounded in its adequacy with the goal of the study.

To illustrate, let us revisit the example of Huntington's disease (Table 2; Figure 3). The dorsal striatum is present in both mice and humans (material similarity). The dorsal striatum controls movements in both mice and humans (functional similarity). In Huntington's disease, neurons undergo cell death and the dorsal striatum degenerates in both murine models and humans (similarity of phenomena at cellular and organ level). Both the murine model and Huntington's patients exhibit motor disorders (similarity of phenomena at behavioral level). The mutation introduced into the mouse genome induces phenomena similar to those observed in human patients (similarity induced by manipulation). These similarities can be interconnected, and in this example, linking the different types of similarities forms the basis of an explanation for Huntington's disease. By considering these cross‐links between similarities, it is possible to avoid validating models based on a list of resemblances that may actually be coincidences. Our distinction in four types of similarities notably facilitates the identification of meaningful and relevant cross‐links for the research project. Recognizing that structure gives rise to a phenomenon, which in turn has a function, enables the coherent connection of similarities.

Three additional remarks can be made regarding the establishment of cross‐links.

#### 4.4.2 Remark 1. A Manipulation‐Induced Similarity Is a Cross‐Link

It is important to note that similarity induced by manipulation is in itself a way to identify cross‐links. The establishment of this similarity suggests the simultaneous establishment of links between similarities of phenomena, structures, or functions, as it links a manipulated structure or phenomenon at one level to a phenomenon at the same or another level. The establishment of cross‐links represents a methodology for the construction of transversal explanations of biological phenomena (valid for several MOs, or valid for both the model and the target), which serve to enhance the external validity of the model.

#### 4.4.3 Remark 2. Mechanistic Explanation Is a Specific Cross‐Link

A specific type of explanation that can reinforce the external validity of a model is a mechanistic explanation. In this type of explanation, multiple material entities are part of a biological mechanism that causally constitutes a phenomenon [106]. These explanations are often considered as the explanatory standards in biology, making them one of the most effective ways to enhance a model's external validity. This is due to at least two reasons. First, elucidating a common mechanism in several MOs, or in both the target and the model, allows us to rule out the possibility of multiple realizations that threaten external validity. When a model has a similar function realized by a different mechanism than in the target, the model is considered to lack external validity. Thus, material similarity alone is insufficient because similar structures can participate in different mechanisms. Second, mechanisms help us identify relevant similarities for the research project. Knowledge of a shared mechanism allows us to determine which physical entities are relevant and should be included in the evaluation. In conclusion, what some authors refer to as “mechanistic validity” is a shared mechanism between several MOs or between the model and the target, which in turn represents a complex link between similarities. Mechanistic explanations correspond to a particular type of cross‐link, which allows researchers to reject purely correlational links and thereby reinforce external validity.

#### 4.4.4 Remark 3. About the Terms Phenomenon and Function

It is also worth noting that assessing external validity through linked similarities remains effective even when researchers interpret the terms “phenomenon” and “function” differently. Some may call “phenomenal similarity” what others may refer to as a “functional similarity,” but this does not affect the overall evaluation of the validity of MOs. However, this distinction retains heuristic value, as it helps to avoid comparing similarities of different types.

### 4.5 Step 5. Integrate All the Elements of the Evaluation

The final step is to take a step back and get the big picture. The framework, a summary of which is provided in Box 5, allows to integrate elements in a structured manner (see Figures 2 and 3) to guide the appropriate use of the model and its possible improvement. This integration combines three complementary elements of conclusions. First, decide whether the model is appropriate for addressing the specific question and level of explanation sought by determining the fit between the model and the research objective. Second, internal validity is synthesized by considering the reliability of the data and the strength of the interpretations. Third, external validity is determined by identifying the most relevant types of similarities, examining their degree, and assessing the coherence of the cross‐links connecting them across biological levels.

This final integrative step also enables the comparison of alternative models. Different MOs may exhibit distinct configurations of internal validity, similarities, and cross‐links. Making these explicit helps to clarify the trade‐offs involved in selecting a model for a given purpose.

## 5 Conclusion

Model organisms play a central and multifaceted role in contemporary biological and biomedical sciences, yet their unique epistemic status often remains underexamined. We have sought to fill this gap by providing a reflexive review as well as a new approach to evaluating the validity of MOs. A key insight emerging from this work is that the biological sciences can benefit from philosophy of science. The proposed evaluative framework illustrates how philosophy of science can clarify concepts and structure critical reflection on experimental methods and scientific assumptions [107].

For instance, establishing cross‐links between the four types of similarities across different biological levels draws on both structure mapping theory in analogical reasoning and mechanistic accounts developed in recent philosophy of science. A central aim is to preserve analytical rigor while avoiding overly abstract concepts. The simple term “cross‐links” was therefore chosen for its heuristic value. It makes the framework accessible and enables biologists to keep their research objectives at the center of the evaluation without compromising the philosophical rigor underpinning the framework.

Another key feature of the framework is its reliance on the distinction between data and phenomenon introduced by philosophers studying the structure of scientific theories. Although existing proposals have advanced our understanding of model validation, they do not fully explain how internal validity shapes external validity. Our framework makes this articulation explicit by incorporating both the reliability of the data and the validity of the inference from data to phenomena—two components of internal validity—into the assessment of the strength of the similarity between MO and its target (external validity).

Overall, this framework is intended as a general, modular, and heuristic tool that generates questions and encourages reflection on the assumptions, limitations, and scope of models. By structuring this process of self‐evaluation, it helps researchers clarify their criteria, identify potential weaknesses, and iteratively refine both experimental designs and their interpretations.

## Author Contributions

H.A. and N.H. designed research, wrote the paper.

## Funding

This work was supported by Sorbonne Université (Sorbonne University).

## Conflicts of Interest

The authors declare no conflicts of interest.

## Data Availability

The authors have nothing to report.
